# Comparison of suture anchor penetration rate between navigation-assisted and traditional shoulder arthroscopic capsulolabral repair

**DOI:** 10.1371/journal.pone.0267943

**Published:** 2022-05-05

**Authors:** Hsiao-Kai Pan, Che-Wei Liu, Ru-Yu Pan

**Affiliations:** 1 Department of Orthopedic Surgery, Wan Fang Hospital, Taipei Medical University, Taipei, Taiwan; 2 Department of Orthopedics, Tri-Service General Hospital, National Defense Medical Center, Taipei, Taiwan; 3 Department of Orthopedics, Cathy General Hospital, Taipei, Taiwan; 4 School of Medicine, National Tsing Hua University, Hsinchu, Taiwan; 5 School of Medicine, College of Medicine, Fu Jen Catholic University, New Taipei City, Taiwan; Assiut University Faculty of Medicine, EGYPT

## Abstract

Proper placement of suture anchors is an important step in Bankart repair as improper placement can lead to failure. Concern surrounding suture anchor placement inspired the use navigation systems in shoulder arthroscopy. We aimed to demonstrate the technological advantage of using the O-arm (Medtronic Navigation, Denver, CO, USA) image guidance system to provide real-time images during portal and anchor placements in shoulder arthroscopy. Consecutive patients (from July to October 2014) who were admitted for arthroscopic capsulolabral repair surgeries were included. Ten patients were randomly enrolled in the navigation group and 10 in the traditional group. The glenoid was divided into four zones, and the penetration rates in each zone were compared between the two groups. In zone III, the most inferior region of the glenoid, the penetration rate was 40.9% in the traditional group and 15.7% in the navigation group (P = 0.077), demonstrating a trend toward improved accuracy of anchor placement with the aid of the navigation system; however, this was not statistically significant. Average surgical time in the navigation and traditional groups was 177.6±40.2 and 117.7±17.6 mins, respectively. American Shoulder and Elbow Surgeons Shoulder Scores showed no difference before and 6 months after surgery. This pilot study showed a trend toward decreased penetration rate in O-arm-navigated capsulolabral repair surgeries and decreased risks of implant misplacement; however, possibly due to the small sample size, the difference was not statistically significant. Further large-scale studies are needed to confirm the possible benefit of the navigation system. Even with the use of navigation systems, there were still some penetrations in zone III of the glenoid. This penetration may be attributed to the micro-motion of the acromioclavicular joint. Although the navigation group showed a significant increase in surgical time, with improvements in instrument design, O-arm-navigated arthroscopy will gain popularity in clinical practice.

## Introduction

Shoulder arthroscopy has been developed and widely applied in Bankart repair and complex capsulolabral repair surgeries. Proper placement of suture anchors is considered an important step in Bankart repair as improper placement can lead to failure [1–3]. Furthermore, a high rate of cortical penetration of inferior anchors has been reported [4, 5]. Concerns surrounding suture anchor placement inspired the use of navigation systems in shoulder arthroscopy.

Computed tomography (CT)-based image guidance technology represents one of the most advanced three-dimensional (3D) navigation systems that is widely used in spine surgery. O-arm (Medtronic Navigation, Denver, CO, USA) provides the advantage of automatic registry with instant intra- and postoperative CT scans, which increases the accuracy of spinal instrumentation [6–8].

The aim of this study was to demonstrate the technological advantage of using the O-arm image guidance system to provide real-time images to assist with portal and anchor placements in shoulder arthroscopy. Our null hypothesis is that participants who receive o-arm navigation arthroscopic capsulolabral repair will show no difference in suture anchor penetration rate, surgical time and American Shoulder and Elbow Surgeons Shoulder Score (ASES) at 6 months follow up from participants in the traditional group.

Although this was a pilot study with a limited number of participants, to the best of our knowledge, this is the first study to use the O-arm in shoulder arthroscopic labral repair. This technology may prove helpful in improving the accuracy of anchor implantation in shoulder arthroscopic labral repair.

## Methods

### Study patients

We recruited and enrolled consecutive patients, who were admitted for capsulolabral repair surgeries from July to October 2014. The study was approved by the Institutional Review Board of Tri-Service General Hospital National Defense Medical Center (approval number: 1-104-04-001). Patients were randomly assigned to the navigation and traditional groups. The method of randomization was block randomization, which randomized participants within blocks such that an equal number were assigned to each treatment. Those who had undergone previous shoulder surgery and those with a history of fracture or any known congenital deformities of the shoulder joint were excluded. All diagnosis were confirmed by MRI and provocative tests for instability: anterior apprehension test for anterior instability; posterior jerk test for posterior instability; inferior sulcus test for inferior instability. There was no postoperative recurrent dislocation at 6 month follow up in both groups. All patients signed a written informed consent form to receive navigated arthroscopy, and the risks of radiological exposure were explained. Patients who disagreed with the random assignment were excluded. The individual pictured in Fig 2 has provided written informed consent (as outlined in PLOS consent form) to publish their image alongside the manuscript.

**Fig 1:**
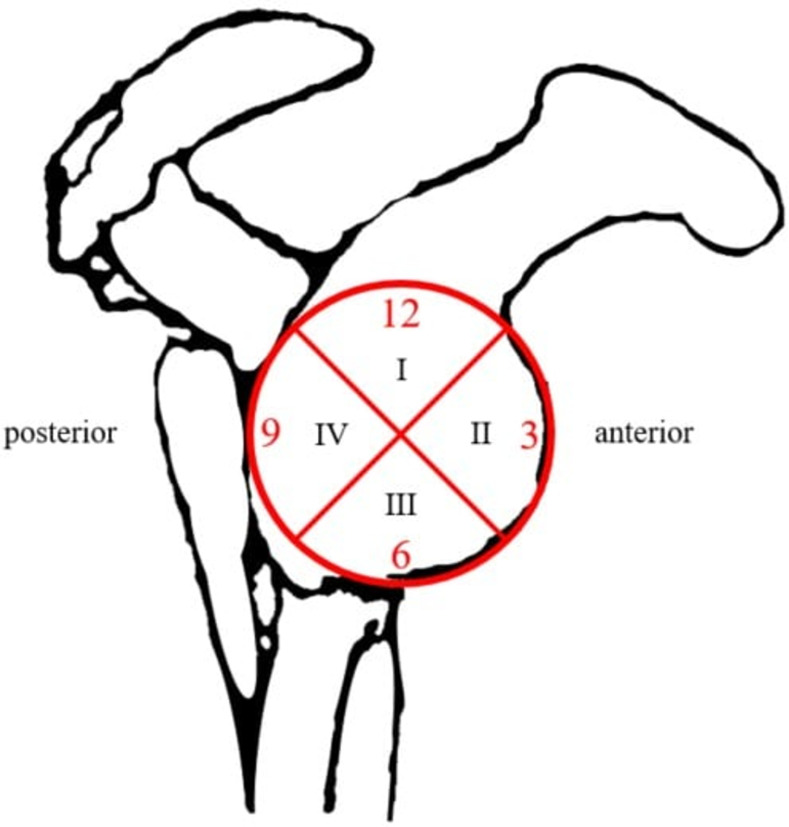
Four zones of the Glenoid (right shoulder). Zone I: 10:30 to 1:30; zone II: 01:30 to 4:30; zone III: 04:30 to 7:30; zone IV: 7:30 to 10:30.

For this study, the glenoid was divided into four zones (Fig 1). Penetration of suture anchors was defined as the protrusion of any part of the anchor over the far cortex. Surgical time was defined as the time between the first skin incision and the end of the last surgical wound suture. Outcome measurements were calculated based on the American Shoulder and Elbow Surgeons Shoulder Score (ASES) before and 6 months after surgery.

### Surgical technique

**Fig 2:**
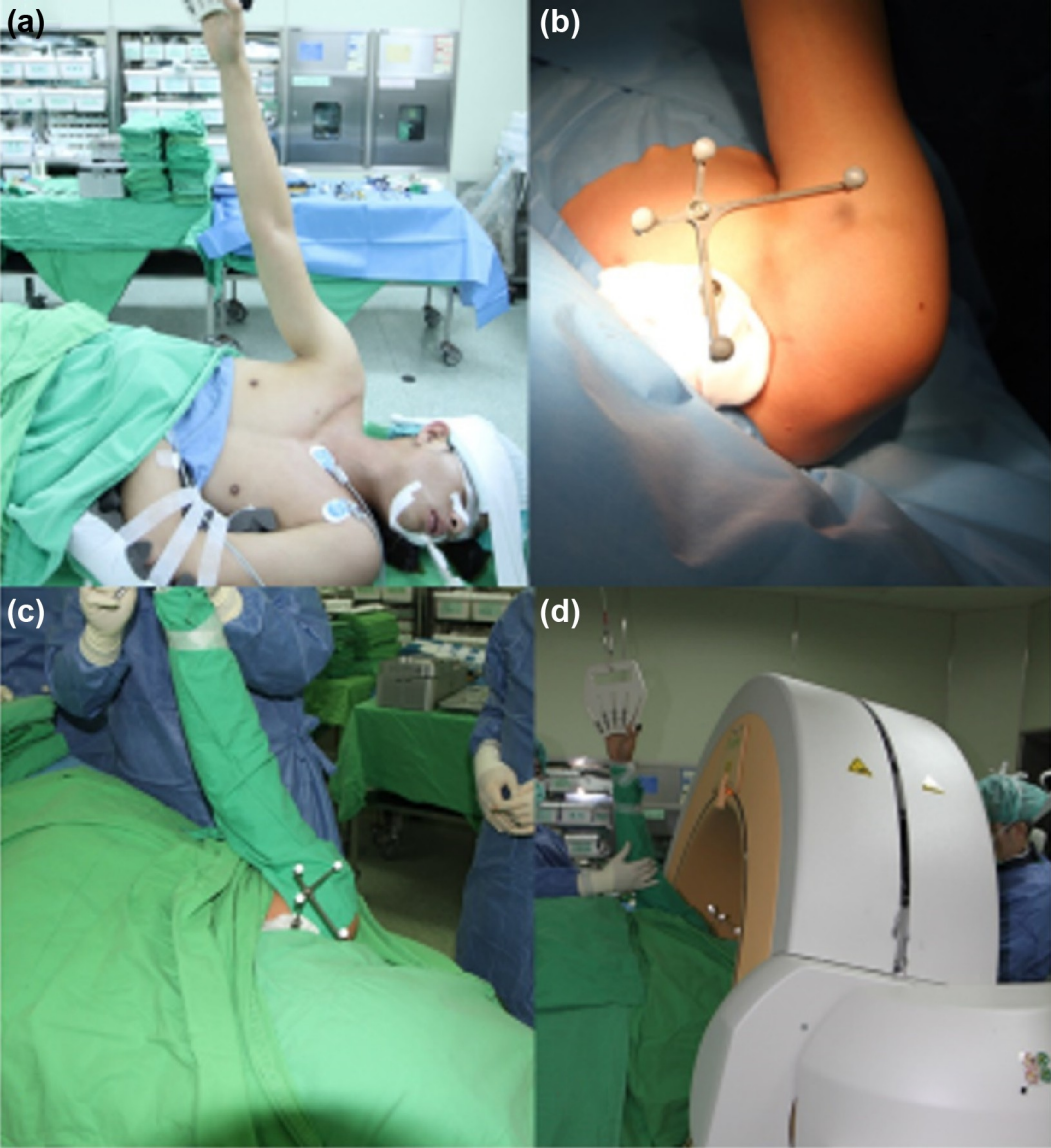
Surgical technique. The patient is in the lateral decubitus position with full support to the abdomen and posterior sacrum (Fig 2a). The shoulder is fixed with an arm sling and suspended with the shoulder retractor. Sterilization and draping are performed as in traditional shoulder arthroscopy surgery. The mid-third clavicle is exposed for fixation of navigation reference frame through a 1-cm incision (Fig 2b). The patient is covered with a sterilized drape (Fig 2c), and the O-arm is positioned around the patient at a 30° tilt to avoid contacting the sterilized arm (Fig 2d).

The patient was positioned in the lateral decubitus position with full support by the abdomen and posterior sacrum (Fig 2A). The shoulder was fixed with an arm sling and suspended with a shoulder retractor. Sterilization and draping were performed as in usual shoulder arthroscopy. The mid-third clavicle was exposed to fix a navigation reference frame through a 1-cm incision (Fig 2B). Our institution utilizes the O-Arm/Stealth Station image-guided navigation platform (Medtronic Navigation, Denver, CO, USA), which is widely used in neurosurgery for pedicle screw instrumentation. The patient was covered with a sterilized drape (Fig 2C), and the O-arm positioned around the patient, tilting at a 30° angle to avoid contact with the sterilized arm (Fig 2D). A CT scan and 3D reconstruction images were obtained and transmitted to the StealthStation (Medtronic, Fridley, Mn, USA). The O-arm was removed, and the arm was suspended with proper traction.

**Fig 3:**
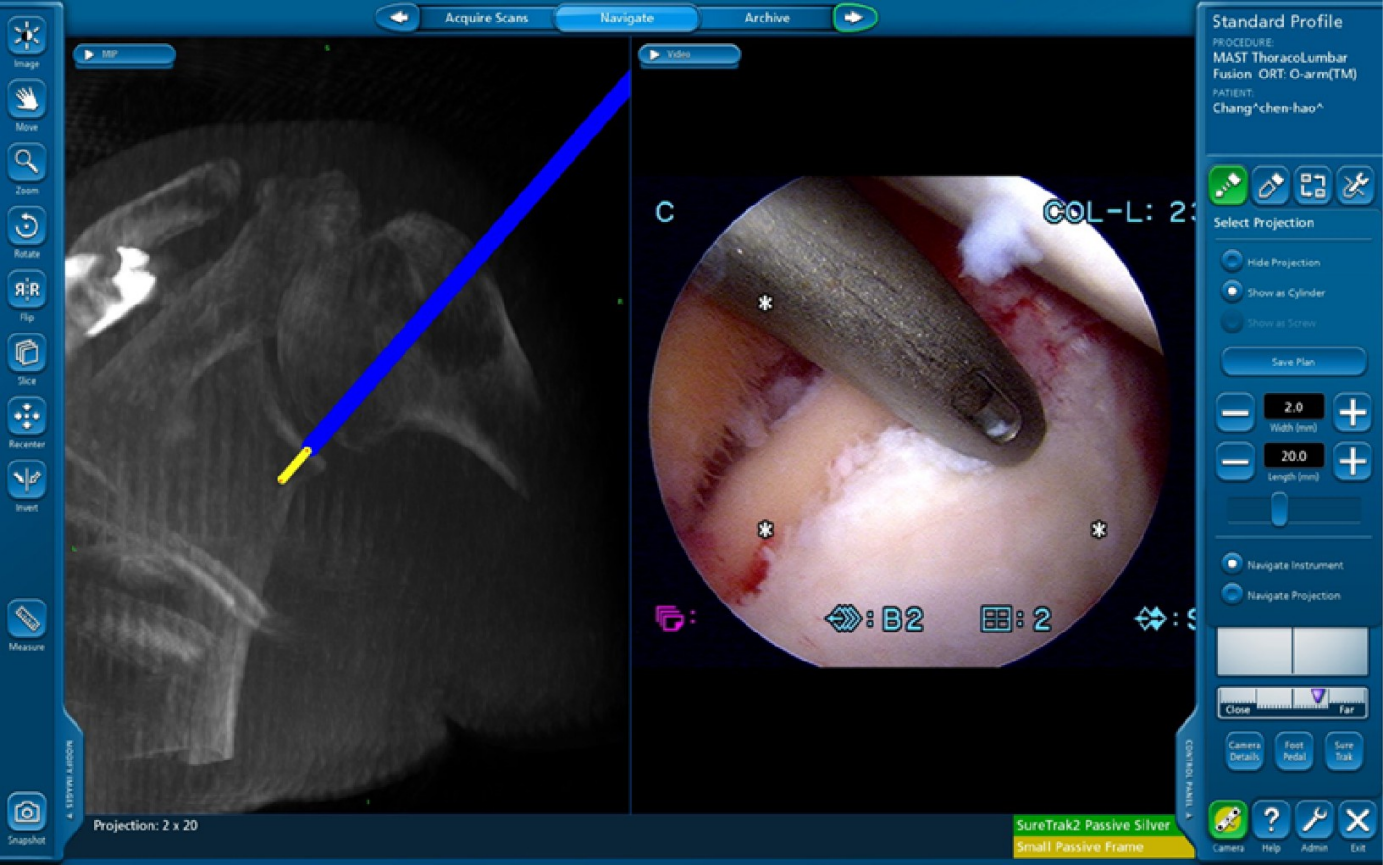
Anchoring and angle of insertion. The depth of the anchors and angle of insertion can be visualized to ensure the best purchase of the suture anchor and to avoid penetration of the contralateral far cortex.

After verifying the basic instruments in the navigation system, the reference frames were set and various registered instruments standardly used in arthroscopy surgery, such as drill guides and suture anchors, were used. For anchor placement, the surgeon may inspect the precise location of the anchor using 3D reconstruction images. The depth of the anchors and the angle of insertion can be visualized to ensure the best purchase of the suture anchor and to avoid penetration of the contralateral far cortex (Fig 3). The type of suture anchors used in the surgery were Smith & Nephew BIORAPTOR 2.9 mm. After surgery, all patients underwent immediate postoperative O-arm scanning for anchor position analysis.

### Statistical analysis

Analyses were performed using SPSS version 17.0 (Chicago, IL, USA). Characteristics of the study population, such as age and body mass index, were described using mean and standard deviations. Penetration rates between the groups were compared using chi-square tests, and odds ratios and 95% confidence intervals were calculated. Surgical times and ASES were compared using the Student’s *t*-test. Statistical significance was set at p-value of <0.05. Detailed clinical information and the underlying data set were provided in supporting information S1 File.

## Results

Twenty-two patients were enrolled to be included in the study. Two patients were excluded due to their refusal to be randomly assigned. Ten patients were randomly assigned to the navigation group and 10 to the traditional group (Table 1). A total of 77 anchor screws were used in the study. The screw locations in the glenoid were divided into four zones as defined previously (Fig 1). Following surgery, all patients underwent immediate postoperative O-arm scanning for anchor position analysis.

**Table 1:** Demography of patients.

	Navigation	Traditional	P-value
**Age (years)**	27.8 ± 4.3	28.4 ± 3.8	0.747
**Sex**	10 males	10 males	
**BMI (kg/m** ^**2**^ **)**	22.62 ± 2.1	22.8 ± 2.1	0.861
**SLAP lesion**	2	0	
**Bankart lesion**	5	6	
**Posterior**	1	2	
**MDI**	2	2	

BMI: body mass index; SLAP: superior labral anteroposterior tear MDI: multi-directional instability

Penetration rates (Table 2) in each zone of the glenoid were compared between the groups. In zone I, the penetration rate was 0% in the navigation group, however, owing to a lack of patients with SLAP lesion in the traditional group, there was no suture anchor placement in this zone, the penetration rate was unable to compare between groups in zone I. In zone II, the rate was 0% in both groups. In zone III, the penetration rate was 40.9% and 15.7% in the traditional and navigation groups, respectively, demonstrating a trend of better accuracy in navigation-assisted anchor placement. However, this difference was not statistically significant (p = 0.077). In zone IV, the penetration rate was 11.1% and 16.6% for the traditional and navigation groups, respectively. This difference was also not statistically significant (p = 0.657).

**Table 2:** Penetration of suture anchors (penetrating screws/total screws).

	I	II	III	IV
**Navigation**	0/2	0/10	3/19 (15.7%)	1/6 (16.6%)
**Traditional**	0/0	0/9	9/22 (40.9%)	1/9 (11.1%)
**P-value**	n.p	n.p	0.077	0.657

The average surgical time in the navigation and traditional groups was 177.6±40.2 and 117.7±17.6 mins, respectively (*p* < 0.001), with a statistically significant difference.

The ASES showed significant improvement after surgery in both groups, but there were no significant differences between the two groups before surgery and 6 months after surgery (Table 3).

**Table 3:** American shoulder and elbow surgeons shoulder scores.

	Navigation	Traditional	P-value
**Before surgery**	65 ± 5.4	66.8 ± 5.2	0.462
**6 Months after operation**	87.2 ± 3.8	85.9 ± 5.2	0.533
**P-value**	<0.0001	<0.0001	

## Discussion

Arthroscopic management of anterior shoulder instability is accepted as a surgical technique with excellent clinical outcomes. Despite advances in implant design and surgical techniques, the risk of recurrence has been reported to be as high as 20%–30% [9–11]. Potential reasons for failure are anatomic variance, technical factors, and patient-related factors [12]. The technical requirements for suture anchor placement include inferior placement of anchors positioned on the glenoid face with an adequate number of anchors and a proper trajectory to achieve stable fixation without surface damage of the articular cartilage [10].

The older generation of navigation systems requires complicated registration procedures, which include pre-operative CT scans and intraoperative structural registration [13]. This complexity makes these older systems difficult to use in shoulder arthroscopy. Our study revealed the feasibility of applying the O-arm navigation system to shoulder arthroscopy.

This study showed a trend of decreased penetration rate in O-arm-navigated capsulolabral repair surgeries and decreased risks of implant misplacement. For the study, the glenoid was divided into four zones. Zone III is located in the most inferior region of the glenoid, which is traditionally the region with the highest penetration rate. This zone had the most benefit from the use of the navigation system. The improvement in accuracy may be attributed to better understanding of the 3D structure of the glenoid and improved self-awareness regarding the angle and depth of the suture anchor.

The ASES improved 6 months after the surgery; however, there was no significant difference in the scores between the navigation and traditional groups. Although penetration of the contralateral far cortex decreases the pull-out strength in cadaveric studies [4, 5], its effects on functional scores are limited.

Even with the use of the navigation system, there were still some penetrations in zone III. This penetration may have been caused by the micro-motion of the acromioclavicular joint. Its micro-motion poses a potential threat to the reference frameshift during navigation. We addressed this problem by decreasing shoulder motion during preparation and by maintaining the traction angle and height as close to the operative status as possible.

Complications have been reported in arthroscopic capsulolabral repair surgeries for suprascapular nerve injuries [14]. The navigation system used in our study can assist surgeons in achieving the desired portal trajectory from the skin to the glenoid and decrease trial and error, theoretically decreasing complications.

Our study revealed that the O-arm navigation system provides intraoperative information on the proper trajectory and possibly more accurate location of the suture anchor on the glenoid. The most inferior anchor in anterior–inferior shoulder instability surgery carries a high rate of far cortical penetration, which may weaken the mechanical strength of the anchor fixation [4, 5]. Using the navigation system, we can project the optimal angle and entry point of the anchors and estimate the optimal depth of anchor penetration to avoid far cortical perforation, theoretically maximizing the pull-out strength of the anchors.

This study has some limitations, which may have affected our results. Since this was a pilot study, only a small number of patients were included, which may account for the lack of statistical significance in zones I, II, and IV. Although there was a trend toward a decreased penetration rate in zone III, it was not statistically significant, possibly due to the small sample size.

In our study, there were more cases of SLAP lesion and less cases of instability in the traditional group, this may be a source of bias since Capsulolabral repair in patients with instability were thought to be more technical demanding than simple SLAP repair and the functional outcome may also be superior in simple SLAP lesions compared to complex instabilities. Unfortunately, our sample size was not big enough to have subgroup analysis regarding different types of capsulolabral injury, therefore further larger scale studies are needed.

The reference point used in this study was mid-clavicle instead of the spine of the scapula or coracoid process. Using scapula as a reference point theoretically may cause less micron-motion since it has a fixed relation to the glenoid. However, the closer proximity of the two reference points makes the operation of the arthroscope difficult. The thickness of the scapular spine and the depth of the coracoid also make the percutaneous fixation of reference points difficult. In future studies, to reduce technical errors, a customed made reference frame specific for shoulder arthroscopy is required for direct fixation on the scapula to form a rigid body for image navigation and to prevent movement in the acromioclavicular joint.

Radiation exposure is also a concern of the use of O-arm navigation system since there’s no radiation in the traditional arthroscopic repair, therefore further studies are needed in the future to find out whether the benefits outweigh the risks.

## Conclusions

There have been few reports on the use of O-arm navigation in shoulder arthroscopy; however, to the best of our knowledge, no reports have specifically addressed labral repair. Our pilot study on shoulder navigation surgery confirmed the applicability of O-arm navigation in shoulder arthroscopic capsulolabral repair. The navigation system provides real-time image guidance for anchor trajectory, location, and depth estimation. Our data demonstrated a trend toward decreased penetration rate (15.7% vs. 40.9%, p = 0.077) in the inferior glenoid in the navigation surgery compared with that in traditional shoulder arthroscopy. However, possibly due to the small sample size, the difference was not statistically significant. Further large-scale studies are needed to confirm the possible benefit of the navigation system in better accuracy of anchor placement. Although the navigation group showed a significant increase in surgical time, with more practice and improvement in future instrument designs, we believe that O-arm-navigated arthroscopy will gain more popularity in clinical practice.

## Supporting information

S1 Data Minimal data set. (XLSX)

## References

[1] Kim SH, Ha KI, Kim YM. Arthroscopic revision Bankart repair: A prospective outcome study. Arthroscopy. 2002;18: 469–482. doi: 10.1053/jars.2002.32230 11987056

[2] Levine WN, Arroy JS, Pollock RG, Flatow EL, Bigliani LU. Open revision stabilization surgery for recurrent anterior glenohumeral instability. Am J Sports Med. 2000;28: 156–160. doi: 10.1177/03635465000280020401 10750990

[3] Sisto DJ. Revision of failed arthroscopic Bankart repairs. Am J Sports Med. 2007;35: 537–541. doi: 10.1177/0363546506296520 17244898

[4] Frank RM, Mall NA, Gupta D, Shewman E, Wang VM, Romeo AA, et al. Inferior suture anchor placement during arthroscopic bankart repair: influence of portal placement and curved drill guide. Am J Sports Med. 2014;42: 1182–1189. doi: 10.1177/0363546514523722 24576744

[5] Lim TK, Koh KH, Lee SH, Shon MS, Bae TS, Park WH, et al. Inferior anchor cortical perforation with arthroscopic Bankart repair: a cadaveric study. Arthroscopy. 2013;29: 31–36. doi: 10.1016/j.arthro.2012.08.013 23276411

[6] Oertel MF, Hobart J, Stein M, Schreiber V, Scharbrodt W. Clinical and methodological precision of spinal navigation assisted by 3D intraoperative O-arm radiographic imaging. J Neurosurg Spine. 2011;14: 532–536. doi: 10.3171/2010.10.SPINE091032 21275555

[7] Silbermann J, Riese F, Allam Y, Reichert T, Koeppert H, Gutberlet M. Computer tomography assessment of pedicle screw placement in lumbar and sacral spine: comparison between free-hand and O-arm based navigation techniques. Eur Spine J. 2011;20: 875–881. doi: 10.1007/s00586-010-1683-4 21253780 PMC3099154

[8] Takahashi J, Hirabayashi H, Hashidate H, Ogihara N, Kato H. Accuracy of multilevel registration in image-guided pedicle screw insertion for adolescent idiopathic scoliosis. Spine (Phila Pa 1976). 2010;35: 347–352. doi: 10.1097/BRS.0b013e3181b77f0a 20075780

[9] Owens BD, DeBerardino TM, Nelson BJ, Thurman J, Cameron KL, Taylor DC, et al. Long-term follow-up of acute arthroscopic Bankart repair for initial anterior shoulder dislocations in young athletes. Am J Sports Med. 2009;37: 669–673. doi: 10.1177/0363546508328416 19218560

[10] Randelli P, Ragone V, Carminati S, Cabitza P. Risk factors for recurrence after Bankart repair a systematic review. Knee Surg Sports Traumatol Arthrosc. 2012;20: 2129–2138. doi: 10.1007/s00167-012-2140-1 22836228

[11] van der Linde JA, van Kampen DA, Terwee CB, Dijksman LM, Kleinjan G, Willems WJ. Long-term results after arthroscopic shoulder stabilization using suture anchors: an 8- to 10-year follow-up. Am J Sports Med. 2011;39: 2396–2403. doi: 10.1177/0363546511415657 21803980

[12] Lehtinen JT, Tingart MJ, Apreleva M, Ticker JB, Warner JJ. Variations in glenoid rim anatomy: implications regarding anchor insertion. Arthroscopy. 2004;20: 175–178. doi: 10.1016/j.arthro.2003.11.029 14760351

[13] Koulalis D, Kendoff D, Citak M, O’Loughlin PF, Pearle AD. Freehand versus navigated glenoid anchor positioning in anterior labral repair. Knee Surg Sports Traumatol Arthrosc. 2011;19: 1554–1557. doi: 10.1007/s00167-010-1360-5 21222107

[14] Morgan RT, Henn RF 3rd, Paryavi E, Dreese J. Injury to the Suprascapular Nerve During Superior Labrum Anterior and Posterior Repair: Is a Rotator Interval Portal Safer Than an Anterosuperior Portal? Arthroscopy. 2014;30: 1418–1423. doi: 10.1016/j.arthro.2014.06.006 25125380

